# The influence of distal screw length on the primary stability of volar plate osteosynthesis—a biomechanical study

**DOI:** 10.1186/s13018-015-0283-8

**Published:** 2015-09-08

**Authors:** Sebastian F. Baumbach, Alexander Synek, Hannes Traxler, Wolf Mutschler, Dieter Pahr, Yan Chevalier

**Affiliations:** Department of Trauma Surgery, University Hospital of Munich (LMU), Campus Innenstadt, Nußbaumstrasse 20, 80336 Munich, Germany; Institute of Lightweight Design and Structural Biomechanics, Vienna University of Technology, Getreidemarkt 9, 1060 Vienna, Austria; Center of Anatomy and Cell Biology, Department of Systematic Anatomy, Medical University Vienna, Währinger Straße 13, 1090 Vienna, Austria; Department of Orthopedic Surgery, Physical Medicine and Rehabilitation, University Hospital of Munich (LMU), Campus Großhadern, Marchioninistrasse 15, 81377 Munich, Germany

**Keywords:** Colles’ fracture, Distal radius fracture, Biomechanics, Volar plate osteosynthesis, Polyaxial volar locking plates, Screw length, Fracture

## Abstract

**Background:**

Extensor tendon irritation is one of the most common complications following volar locking plate osteosynthesis (VLPO) for distal radius fractures. It is most likely caused by distal screws protruding the dorsal cortex. Shorter distal screws could avoid this, yet the influence of distal screw length on the primary stability in VLPO is unknown. The aim of this study was to compare 75 to 100 % distal screw lengths in VLPO.

**Methods:**

A biomechanical study was conducted on 11 paired fresh-frozen radii. HRpQCT scans were performed to assess bone mineral density (BMD) and bone mineral content (BMC). The specimens were randomized pair-wise into two groups: 100 % (group A) and 75 % (group B) unicortical distal screw lengths. A validated fracture model for extra-articular distal radius fractures (AO-23 A3) was used. Polyaxial volar locking plates were mounted, and distal screws was inserted using a drill guide block. For group A, the distal screw tips were intended to be flush or just short of the dorsal cortex. In group B, a target screw length of 75 % was calculated. The specimens were tested to failure using a displacement-controlled axial compression test. Primary biomechanical stability was assessed by stiffness, elastic limit, and maximum force as well as with residual tilt, which quantified plastic deformation.

**Results:**

Nine specimens were tested successfully. BMD and BMC did not differ between the two groups. The mean distal screw length of group A was 21.7 ± 2.6 mm (range: 16 to 26 mm), for group B 16.9 ± 1.9 mm (range: 12 to 20 mm). Distal screws in group B were on average 5.6 ± 0.9 mm (range: 3 to 7 mm) shorter than measured. No significant differences were found for stiffness (706 ± 103 N/mm vs. 660 ± 124 N/mm), elastic limit (177 ± 25 N vs. 167 ± 36 N), maximum force (493 ± 139 N vs. 471 ± 149 N), or residual tilt (7.3° ± 0.7° vs. 7.1° ± 1.3°).

**Conclusion:**

The 75 % distal screw length in VLPO provides similar primary stability to 100 % unicortical screw length. This study, for the first time, provides the biomechanical basis to choose distal screws significantly shorter then measured.

**Electronic supplementary material:**

The online version of this article (doi:10.1186/s13018-015-0283-8) contains supplementary material, which is available to authorized users.

## Background

Recent studies have reported complication rates following volar locking plate osteosynthesis (VLPO) for distal radius fractures of up to 18 % [1, 2]. Two of the most common complications are extensor tendon irritation and attritional tendon ruptures [1, 3, 2]. These are attributable either to direct damage during the operation (drilling, depth gauge) or secondary due to dorsodistal screw protrusion [4–6].

Dorsal screw protrusion might be an avoidable complication, especially for extra-articular fractures (AO-23 A3), which are the most common ones [7, 8]. The AO Foundation [9] as well as *Campbell’s Operative Orthopaedics* [10] recommends using distal screw length 2 to 4 mm shorter than measured. However, the effect of shorter distal screws on the primary stability of the VLPOs remains unclear. Preliminary data on synthetic bones indicates that 75 % distal screw length provides comparable primary stability to 100 % unicortical screw length [11].

Shorter distal screws are the most promising approach to avoid dorsal screw protrusion. Therefore, it is indispensable to investigate the effect of distal screw length on the primary stability of VLPO. Consequently, the aim of this study was to compare 75 to 100 % distal screw lengths in VLPO using human fresh-frozen radii and an established biomechanical fracture model for extra-articular distal radius fractures (AO-23 A3). The study’s null hypothesis was that unicortical 100 % distal screw lengths provide superior primary stability compared to 75 % distal screw lengths in VLPO.

## Methods

This biomechanical study was conducted on fresh-frozen human radii using a validated fracture model for extra-articular distal radius fractures (AO-23 A3). The local ethics committee approved the study (LMU #409-13). The outcome parameters of interest were stiffness, elastic limit, maximum force, and residual tilt of the distal fragment.

Eleven paired fresh-frozen radii were obtained from the Centre of Anatomy and Cell Biology, Medical University of Vienna, Austria. Radii were randomized pair-wise, side alternating into a 100 % unicortical distal screw length group (group A) and a 75 % distal screw length group (group B). They were then cut to 14-cm length. High-resolution peripheral quantitative computer tomography scans (HRpQCT, XtremeCT, Scanco Medical AG, Switzerland) were performed. Radii presenting previous fractures, severe osteoarthritis, or bone lesions were excluded. Bone mineral density (BMD) and bone mineral content (BMC) were computed [12] to assess possible group differences.

### Specimen preparation

**Fig. 1:**
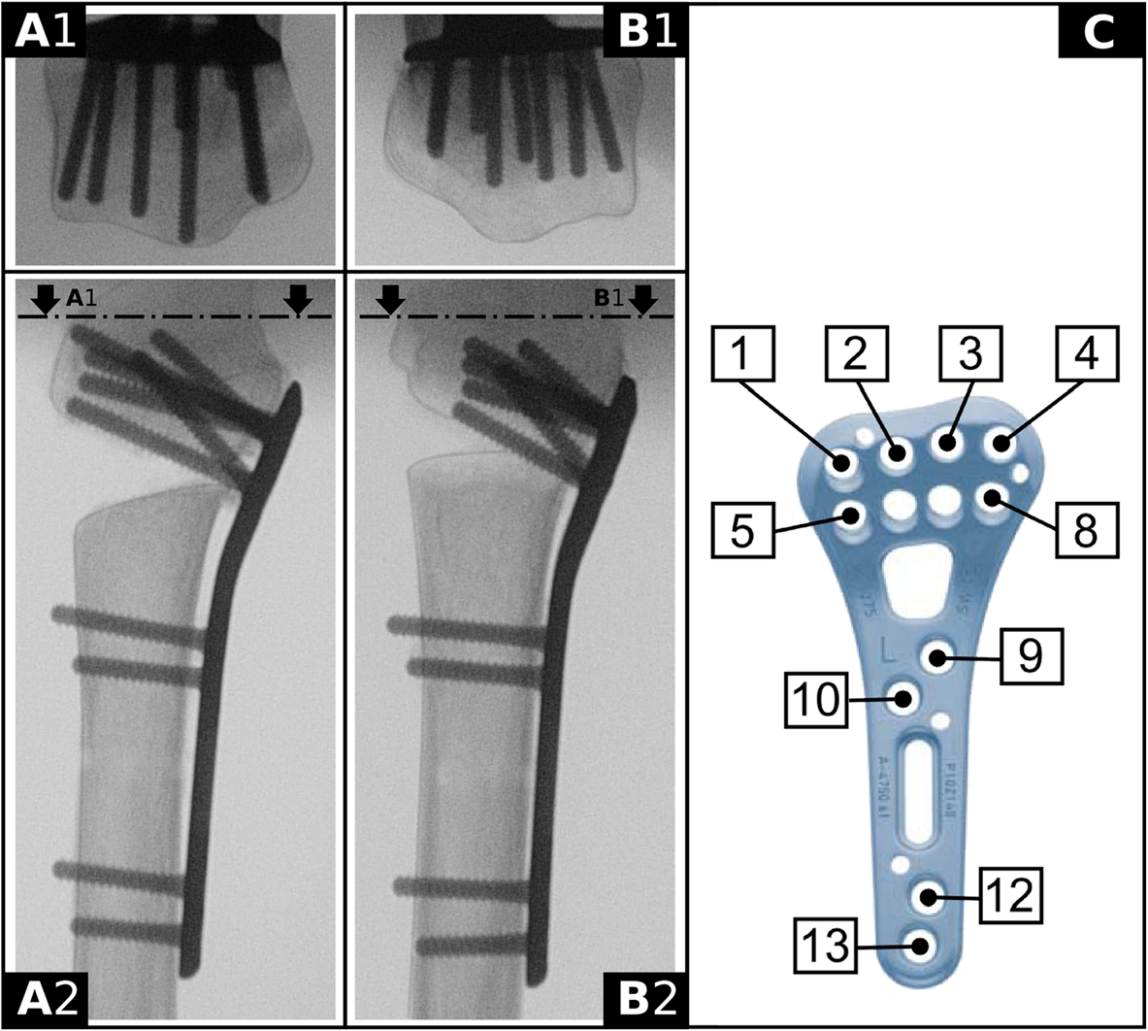
Outline of the 100 % (group A) and 75 % distal screw length (group B) setups. **A** unicortical, 100 % distal screw length (Group A). **B** 75 % distal screw length (Group B). *1* Skyline view, *2* lateral radiograph

The general preparation has been outlined in detail previously [13]. In brief, the radii were cleaned of all soft tissue and multidirectional, angular stable volar plates (APTUS 2.5 ADAPTIVE TriLock Distal Radius Plate, A-4750.61, Medartis Inc., Basel, Switzerland) were mounted just proximal to the watershed line. The plates were fixed to the radius shaft using four bicortical locking screws (Fig. 1C, screws 9, 10, 12, and 13).

A drill guide block (Medartis A-2723 01/02) was mounted onto the distal plate, which assured uniform distal screw orientation. Following drilling, distal screw length was measured. Distal locking screw lengths were chosen according to the previously defined groups. For group A (100 %), the screw tips were intended to be flush or just short of the dorsal cortex (Fig. 1A). In group B (75 %), a target screw length of 75 % was calculated and rounded to the next available screw length (Fig. 1B, C, screws 1–5 and 8). Screws were available in 2-mm increments.

Following distal screw insertion, a 10-mm dorsal wedge osteotomy simulating a dorsally unstable fracture was performed using an oscillating handsaw. The osteotomy location resembled the in vivo fracture location and was chosen based on previous studies [14, 13]. Care was taken to completely separate the volar cortex (1-mm gap).

**Fig. 2:**
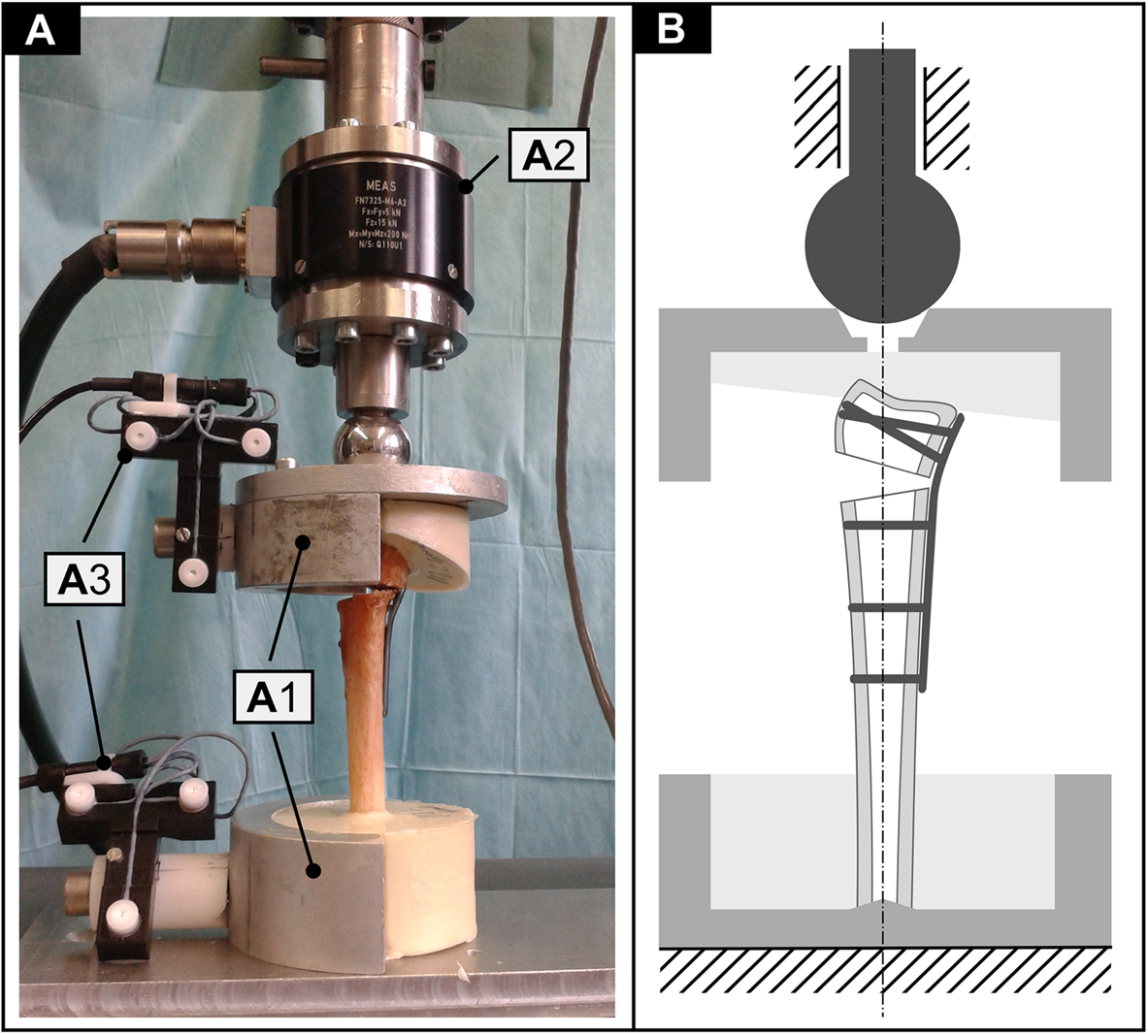
Illustration of the biomechanical setup (one half of the aluminium jigs were removed to show the embedding). **A** Photography of the final setup; *A1* custom-made aluminium-jigs, *A2* multiaxial load cell, *A3* Zebris motion tracking system. **B** Schematic drawing of the final setup illustrating the load axis

Each specimen was then embedded using two custom-made aluminium jigs. The load axis was defined proximally by the medullary canal and distally slightly dorsoradial to the centre of the crista subdividing the fossa lunata and scaphoidea. The proximal 40 mm of the shaft and a shallow edge of the distal articular surface of the radii were embedded in polyurethane (PUR, FDW HG, Austria) (Fig. 2B).

### Biomechanical testing

A proximal constrained setup was used (Fig. 2). The embedded specimens were remounted to the aluminium jigs (Fig. 2A(A1)) and aligned within the material testing machine (Fig. 2A(A2); Zwick-Modell Z010/TN2A; Zwick GmbH & Co. KG, Ulm, Deutschland). Load was applied distally through a 32-mm metal sphere, which enabled free rotation of the distal fragment. It was centred in a centring bore to ensure consistent loading conditions (Fig. 2B). Three markers of a CMS20S ultrasound motion tracking system (Zebris Medical GmbH, Isny im Allgäu, Germany) were mounted to measure residual tilt of the distal fragment (Fig. 2A(A3)).

Specimens were tested to failure using a displacement controlled axial compression test. Following preconditioning to exclude settling effects (preload: 10 N; preconditioning: 10 cycles, 0.2mm displacement, 1 mm/s), the specimens were loaded at 1 mm/s until either a 20 % force drop or 3mm displacement was reached [13, 15]. Photographs and radiographs were taken before and after testing.

### Data analysis

**Fig. 3:**
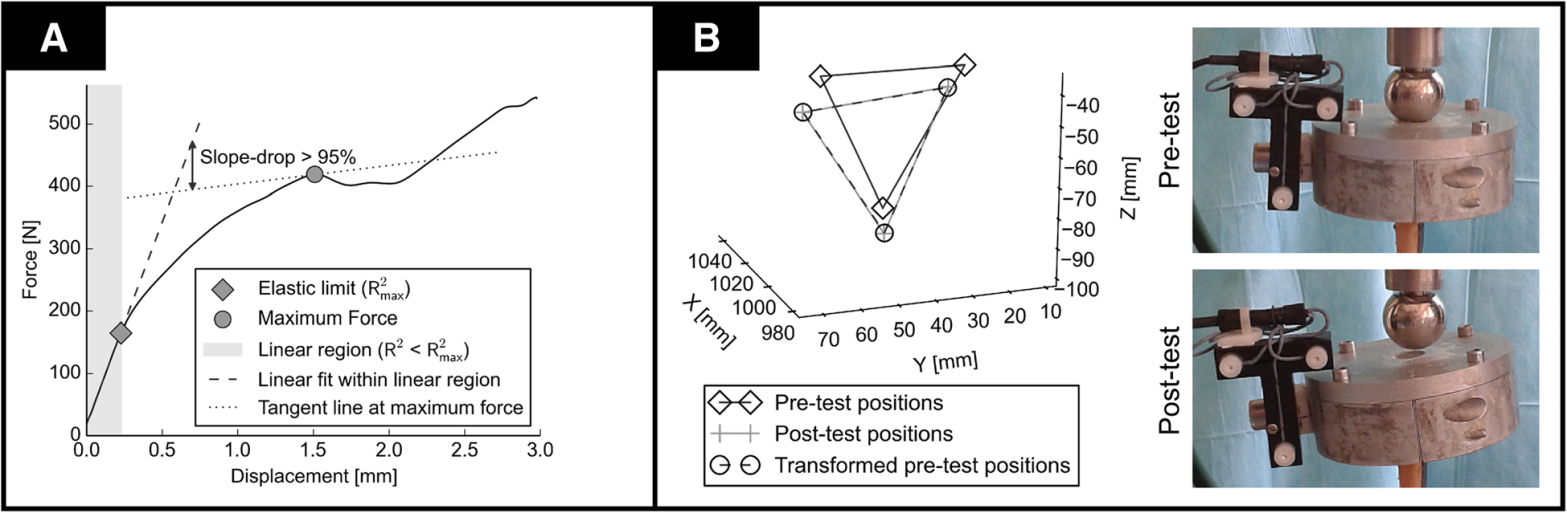
Analysis of the load-displacement curves and the motion tracking system. **A** Analysis of a typical force-displacement curve. **B** Analysis of the residual tilt using the Zebris motion tracking system

Primary biomechanical stability was assessed by stiffness, elastic limit, and maximum force. These were calculated from the load-displacement curves. Data analysis was conducted automatically in Python using custom scripts as outlined in Fig. 3a. The elastic range was defined as the data range until the coefficient of determination reached its maximum (*R*^2^ > 0.998). The elastic limit corresponded the last data point of the elastic range. Stiffness was defined as the slope of the regression line within the elastic range. Maximum force was defined as the force where the slope of the tangent line dropped below 95 % of the stiffness. In one case, the slope did not reach this threshold and the global maximum force was chosen. Residual tilt was determined using the motion tracking system to quantify the overall plastic deformation. It was defined as the angle between the initial and final testing position of the distal jig and assessed by rigid registration of the initial and final marker positions (Fig. 3b).

### Statistics

In addition to standard descriptive statistics, independent sample *t* tests were conducted for all biomechanical parameters. Normality and equality of variances for those parameters were tested using the Shapiro-Wilk and *F* test, respectively. Screw length measurements were not normally distributed and analysed using the Mann-Whitney *U* test. A Bonferroni correction was applied with an adapted level of significance of 0.0125 to account for multiple testing.

## Results

Two specimens were excluded, one due to previous fracture and one because of misalignment during testing. The mean age of the remaining nine pairs was 85.6 ± 11.1 years. Four donors were female. BMD and BMC did not differ between the two groups.

Table 1 shows distal screw length details and statistics for each distal screw separately. Distal screw lengths were significantly greater in group A (21.7 ± 2.6 mm; range: 16 to 26 mm) compared to group B (16.9 ± 1.9 mm; range: 12 to 20 mm). In group B, screws were on average chosen 5.6 ± 0.9 mm (range: 3 to 7 mm) shorter than measured.

**Table 1:** Details on measured and chosen distal screw length [mm]

Number of screw^a^	Parameter	Group A 100 %	Group B 75 %	Significance^b^
1	Measured	20.3 ± 2.6	19.7 ± 2.7	ns
	Chosen	19.1 ± 2.7	15.1 ± 2.0	0.003
	Percent total [%]	93.9 ± 3.7	76.7 ± 2.8	–
2	Measured	23.6 ± 1.5	24.1 ± 1.8	ns
	Chosen	22.7 ± 1.4	18.0 ± 1.4	<0.001
	Percent total [%]	96.0 ± 3.0	74.8 ± 2.3	–
3	Measured	23.9 ± 2.0	23.6 ± 2.4	ns
	Chosen	23.3 ± 2.4	17.8 ± 1.9	<0.001
	Percent total [%]	97.3 ± 2.6	75.3 ± 1.9	–
4	Measured	23.3 ± 2.0	23.1 ± 1.7	ns
	Chosen	22.4 ± 2.2	17.1 ± 1.1	<0.001
	Percent total [%]	96.1 ± 3.0	74.1 ± 2.0	–
5	Measured	21.3 ± 2.7	21.3 ± 2.0	ns
	Chosen	20.2 ± 2.3	16.0 ± 1.7	<0.001
	Percent total [%]	94.9 ± 3.0	75.1 ± 2.8	–
8	Measured	23.1 ± 1.8	23.3 ± 2.2	ns
	Chosen	22.2 ± 2.3	17.6 ± 1.7	<0.001
	Percent total [%]	96.0 ± 4.1	75.2 ± 2.2	–

Values given in millimetre if not stated differently

*ns* not significant

^a^According to Fig. 1C

^b^Mann-Whitney *U* test

The analysis of the biomechanical outcome parameters revealed no differences between the 75 and 100 % distal screw length group for any parameter (Table 2). Therefore, the null hypothesis had to be rejected. Additional comparison between left and right as well as female and male radii revealed no significant differences for all parameters except a greater residual fragment tilt in female specimen (7.9 ± 0.8° vs. 6.6 ± 0.7°; *p* = 0.001).

**Table 2:** Biomechanical results comparing 75 to 100 % distal screw lengths

Parameter	Parameter	Group	Mean	SD	Sig.
Load-displacement curves	Stiffness [N/mm]	A (100 %)	706	103	0.412
		B (75 %)	660	124	
	Elastic limit [N]	A (100 %)	177	25	0.496
		B (75 %)	167	36	
	MaxForce [N]	A (100 %)	493	139	0.750
		B (75 %)	471	149	
Zebris	Residual tilt [degree]	A (100 %)	7.3	0.7	0.755
		B (75 %)	7.1	1.3	

MaxForce: Maximum force as defined as the force when the slope of the tangent line dropped below 0.95 % of the stiffness; A: 100 % unicortical distal screw length (group A); B: 75 % distal screw length (group B)

*SD* standard deviation, *Sig.* adjusted (Bonferroni) level of significance 0.0125

## Discussion

Extensor tendon irritation and attritional tendon ruptures are two of the most common complications following VLPO. Both can be caused by distal screws protruding the dorsal cortex [4–6]. Shorter distal screws can preclude dorsal screw protrusion [9, 10]. This biomechanical study demonstrated that 75 % distal screw lengths provides similar primary stability to 100 % screw lengths in a cadaver model.

The authors are only aware of two studies, investigating the effect of distal screw length on the primary stability of VLPO, both with inherent limitations. Greenberg et al. [16] presented an abstract at the Annual Meeting of the AAOS comparing three different distal screw lengths: 75 %, 100 % unicortical, and bicortical. Three fresh-frozen radii were tested per group. No details were given on the biomechanical setup. No group differences were found. The small sample size and the missing information on the setup hinder data interpretation. Wall et al. [11] compared 50, 75, and 100 % unicortical distal screw lengths in synthetic radii. No significant differences between 100 and 75 % distal screw length were reported. However, these conclusions are limited due to the use of synthetic radii in an inadequate fracture model.

In general, the validity of a biomechanical study relies on the test setup used. We tried to apply a best-evidence setup based on previous experiments and literature [15, 13]. Previous setups vary in almost every aspect, i.e. boundary conditions, the fracture model, and the specimens used [17–20]. All of these have a pronounced impact on the biomechanical outcome parameters. One of these varying parameters is the location of the osteotomy mimicking dorsally unstable distal radius fractures. Its impact on the biomechanical outcome parameters has been highlighted recently [13]. Wall et al. [11] removed a 10-mm dorsal wedge based 10 mm proximal to Lister’s tubercle [21, 19, 18]. Previous studies have removed similar sized wedges 10 to 25 mm proximal to the articular surface [22–26]. The herein applied standardized fracture model [15, 13] bases the osteotomy location on a radiographic study, which has analysed the in vivo distal fracture location in distal radius fractures [14]. We believe that the use of a standardized fracture model [15, 13] is a strength of our study. Another decisive parameter for the validity of a biomechanical study is the type of specimen tested. Wall et al. [11] chose a sawbone model (#1027-130, Sawbones; Pacific Laboratories Inc., Vashon, WA, USA), which, although applied in previous studies [27, 21, 28], is not recommended for biomechanical testing by the manufacturers as it does not replicate structural properties of bone. Moreover, a previous study reported a significantly different biomechanical behaviour compared to fresh-frozen radii [13]. Consequently, the use of paired fresh-frozen radii is another strength of this study. A further advantage is the use of paired samples, which allows pair-wise, side-alternating randomization. This ensures a high homogeneity for morphometric and structural parameters.

The results of our study are corroborated by comparison to literature. As outlined above, the biomechanical setups published for distal radius fractures vary significantly. This not only alters the biomechanical behaviour of the mode, which consequently leads to diverging results, but also hampers inter-study comparison. Still, similar maximum force values were reported in previous studies applying a comparable setup [19, 29]. Moreover, the herein observed maximum force values exceeded 250 N for both groups, which is usually considered the maximum force occurring during rehabilitation [30–32].

Although various biomechanical parameters associated to failure of the osteosynthesis have been assessed, the actual failure mode has not. Possible failure modes include screw-bone, screw-plate, or plate failure. These could be influenced by distal screw length. First, shorter screws reduce the screw-bone contact area, which might increase the local damage around the screws during loading and therefore influence total plastic deformation. In this study, residual tilt was chosen as a surrogate parameter to quantify total plastic deformation [33]. Other studies attempted to quantify residual deformation by  the displacement at the fracture gap [34] or along the loading axis [35]. Both parameters are considered less reliable than residual tilt due to their dependence on the specimen's geometry. The herein observed gender differences could be associated to gender differences in BMC or bone geometry. Second, shorter distal screws reduce the screws’ lever arm acting on the plate. This could have an impact on the screw-plate interface. Screw-plate failure, i.e. screw push-out, is a known complication following polyaxial VLPO [36, 37]. To our best knowledge, no biomechanical study has yet analysed this failure mode. In order to get a first insight, we conducted pre- and post-testing lateral radiographs and photographs to visually evaluate screw push-out (Additional file 1). For group A, five screw push-outs (screws 1 (×1), 5 (×2), 8 (×2)) occurred in three specimens. For group B, two screw push-outs (screws 5 (×2)) occurred in two specimens. Still, screw-plate failure is not only influenced by screw length, but by various parameters, including screw orientation and bone quality. Computational analyses are needed to assess the actual load distribution within the screw-plate construct. This would help to optimize the actual load distribution and thereby increase the construct’s overall stability.

A further limitation might be the used axial loading protocol, as it does not account for all loading conditions during early rehabilitation. Although few authors conducted specific bending and torsion tests [38], most biomechanical distal radius fracture studies applied axial compression testing. Constrained axial compression also results in considerable shear forces and moments and is therefore believed to simulate all relevant forces occurring within the construct [39, 40]. Moreover, while some studies applied fatigue testing [39, 11], our goal was the assessment of primary stability, following previous studies [34, 17, 13]. Finally, the influence of distal screw length was only assessed for the most common distal radius fracture (AO-23 A3) using a biomechanical fracture model. Whether this concept can be adapted to fractures in vivo and intra-articular distal radius fractures (AO-23 C) has yet to be evaluated.

## Conclusion

This biomechanical study was able to demonstrate that 75 % distal screw length can provide similar primary stability as unicortical 100 % distal screw length in VLPO. This study, for the first time, provides the biomechanical basis to choose distal screws significantly shorter then measured. Future clinical studies are required to validate this approach in vivo and investigate on the possible reduction of dorsal screw protrusion incidences and subsequent extensor tendon problems.

## Additional file

Additional file 1:
**Illustration of screw push-out (black arrows).** A) Specimen prior to testing; B) specimen after testing; 1) photographs; 2) radiographs.

## Abbreviations

#: number
%: percent
×: times
°: degrees
AO-23 A3: dorsally displaced extra-articular distal radius fractures
AO-23 C: dorsally displaced intra-articular distal radius fractures
BMC: bone mineral content
BMD: bone mineral density
HRpQCT: high-resolution peripheral quantitative computer tomography
mg HA: milligramme hydroxyapatite
mm: millimetre
mm/s: millimetre per second
N: Newton
N/mm: Newton per millimetre
*R*^2^: coefficient of determination
VLOP: volar locking plate osteosynthesis
