# Supplementary figures and images for: The influence of distal screw length on the primary stability of volar plate osteosynthesis—a biomechanical study
Source: J Orthop Surg Res. 2015 Sep 8;10:139. doi: 10.1186/s13018-015-0283-8 (PMC4563846; doi:10.1186/s13018-015-0283-8)

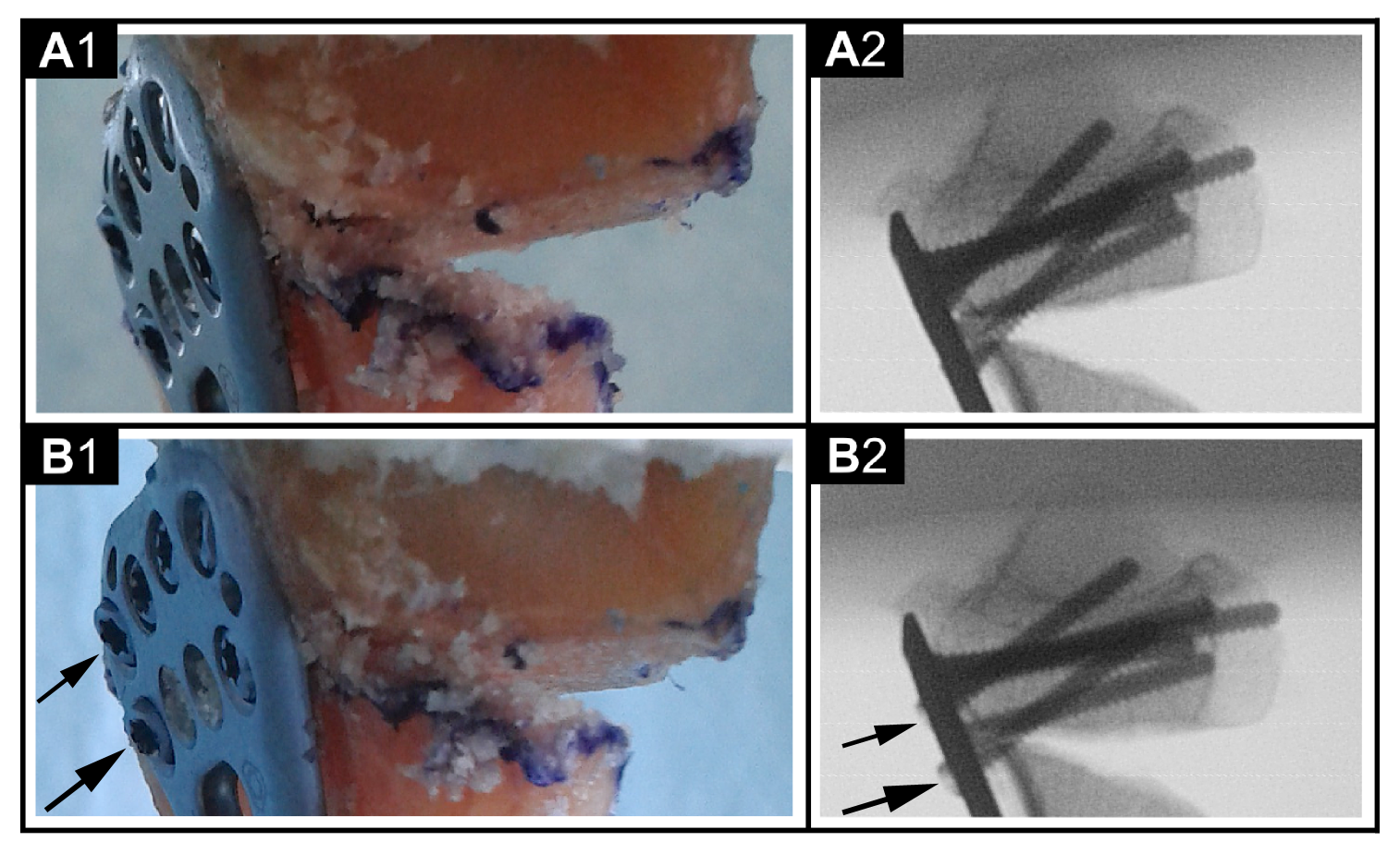

Supplement: Additional file 1: — Illustration of screw push-out (black arrows). A) Specimen prior to testing; B) specimen after testing; 1) photographs; 2) radiographs. [file 13018_2015_283_MOESM1_ESM.tif]
